# Effect of multiple short highly energetic X-ray pulses on the synthesis of endoglucanase by a mutant strain of *Trichoderma reesei*-M7

**DOI:** 10.1080/13102818.2014.966543

**Published:** 2014-10-31

**Authors:** Orlin Gemishev, Stanislav Zapryanov, Alexander Blagoev, Maya Markova, Valentin Savov

**Affiliations:** ^a^ Department of Biotechnology, Faculty of Biology, Sofia University ‘St. Kliment Ohridski’, Sofia, Bulgaria; ^b^ Department of Optics and Spectroscopy, Faculty of Physics, Sofia University ‘St. Kliment Ohridski’, Sofia, Bulgaria

**Keywords:** endoglucanase, X-ray pulses, thermoluminescent dosimeters (TLD), dense plasma focus (DPF), *Trichoderma reesei*

## Abstract

Bioconversion of cellulose-containing substrate to glucose represents an important area of modern biotechnology. Enzymes for the degradation of the polysaccharide part of biomass have been produced, mostly by fungi belonging to genus *Trichoderma*. Studies were carried out with the mutant strain *Trichoderma reesei-*M7, a cellulase producer. Spores of the enzyme producer were irradiated with different doses of characteristic X-ray radiation from metallic tungsten (mainly the W Kα1 and Kα2 lines) with a high dose rate. The latter is a specific property of the dense plasma focus (DPF) device, which has pulsed operation and thus gives short and highly energetic pulses of multiple types of rays and particles. In this case, we focused our study on the influence of hard X-rays. The doses of X-rays absorbed by the spores varied in the range of approximately 5–11,000 mSv measured with thermoluminescent dosimeters (TLD). The influence of the applied doses in combination with exceptionally high dose rates (in the order of tens of millisieverts per microsecond) on the activity of the produced endoglucanase, amount of biomass and extra-cellular protein, was studied in batch cultivation conditions. In the dose range of 200–1200 mSv, some enhancement of endoglucanase activity was obtained: around 18%–32%, despite the drop of the biomass amount, compared with the untreated material.

## Introduction

Bioconversion of cellulose-containing substrate to glucose represents an important area of modern biotechnology. Enzymes for the degradation of the polysaccharide part of biomass have been produced, mostly by fungi belonging to genus *Trichoderma*. One such enzyme is endo-1,4-β-glucanase.

Dense plasma focus (DPF) devices, as the one used in this study, transform the energy of a condenser bank into the energy of a rapidly moving current sheath consisting of highly ionized plasma. The acceleration phase is followed by compression of the current sheath and formation of a small cylindrical column consisting of dense magnetized plasma (pinch). In the pinch period, at conditions specific for each particular device, a powerful flux of X-rays and beams of charged particles appears (reviewed in [1,2]).

Despite the considerable period of study of the discharge in this geometry, there is still an interest towards the phenomenon due to some unresolved problems of the discharge development and dynamics (for example, breakdown phase) and the resources for different applications of the plasma focus (PF) machines. One of these applications is the study of the impact of radiation emitted by the PF discharge on live microorganisms, living cells or other biological objects. There are a few similar works, although PF radiation has some advantages over the conventional ionization sources. The DPF discharge ensures, in the pinch phase, short (3 × 10^−8^ to 10^−7^ s) and powerful X-ray and particle pulses. The influence of soft X-rays or extreme vacuum ultraviolet (VUV) radiation on living cells or microorganisms is not yet well quantitatively studied. On the other hand, it was shown that in the dose range of about 11–65 mSv mainly soft X-radiation can produce a considerable change in the vital characteristics of microalgae (*Chlamydomonas reinhardtii*).[3]

PF discharge is promising for such studies since it generates powerful but short pulses of radiation in this spectral region. In [4–6], medium-energy and hard X-rays of the DPF discharge have been used for the needs of radiation enzymology. Different enzymes have been irradiated *in vitro* at different conditions, e.g. X-ray spectral range, doses, dose power, etc. The reported results show huge differences in the enzyme activation/deactivation after its irradiation by pulsed X-rays (bursts) from PF and from a conventional continuous radioisotope source (γ-source ^137^Cs) and X-ray tube. In particular, the above-mentioned effect is seen at doses five to six orders of magnitude lower for this radiation source than in the case of prolonged action of hard radiation having low intensity. The authors presume that the most adequate characteristic of the action is not the dose itself, but the product of the dose and the dose rate.

Free-living fungi, such as *Trichoderma* spp., are very common in soil and root ecosystems. In this study, spores of strain *Trichoderma reesei*-M7 were irradiated with hard X-ray radiation in the energy range of approximately 0.1–120 keV. Absorbed doses were within the range of approximately 5–11000 mSv.

## Materials and methods

### Strain and cultivation conditions

The investigated *T. reesei* strain M7 (overproducer of celluase enzymes) was obtained by mutagenesis with nitroso-guanidine from the parent strain *Trichoderma* sp. 914.[7] The experiment was conducted with a 10-day-old culture of *T. reesei* strain M7 grown on potato-dextrose agar (PDA) at 28 °C, or a spore suspension (with a density of 5–7 × 10^6^ CFU/mL) prepared in saline solution.

After irradiation, inoculums were obtained in 500 mL flasks which contained 100 mL of the Mandels mineral salt medium [8] with 2% glucose added and 1% maize extract (starting pH after autoclaving 4.8–5.0) at 28 °C with constant shaking (220 r/min) for 24 h. The fermentation mixture (50 mL) was composed of Mandels mineral salt medium with added 1% microcrystaline cellulose Micricel® and 1% wheat bran (starting pH after autoclaving 5.8–5.9). The fermentation process was carried out in triplicates at 28 °C with constant shaking (220 r/min). Endo-1,4-β-glucanase (Cx) activity was measured at the 120th hour of the cultivation process.

### Endo-1,4-β-glucanase activity

Endo-1,4-β-glucanase activity was detected with sodium carboxy-methyl cellulose (Na-CMC) as a substrate, according to Wood and Bhat [9]. The reaction mixture containing 0.5 mL of 1% Na-CMC solution in 0.05 mol/L sodium-acetate buffer (pH 4.8) and 0.5 mL enzyme solution was incubated at 50 °C for 30 min.

Samogyi–Nelson assay [10] was used to assess the level of reducing sugars. One unit of activity was defined as the amount of enzyme that released 1 μmol glucose per minute at the experimental conditions (50 °C, pH 4.8).

### Total protein content and biomass

Total protein content (all soluble proteins) was determined at the 120th hour of the batch (submerged) cultivation by the method of Bradford, using bovine serum albumin as a standard.[11] All measurements were made in triplicates and the standard error was calculated for the three separate samples.

Biomass was measured after 24 h of batch cultivation by determining the biomass dry weight (mg/mL) of vegetative inoculums.

### X-ray irradiation

**Figure 1.**
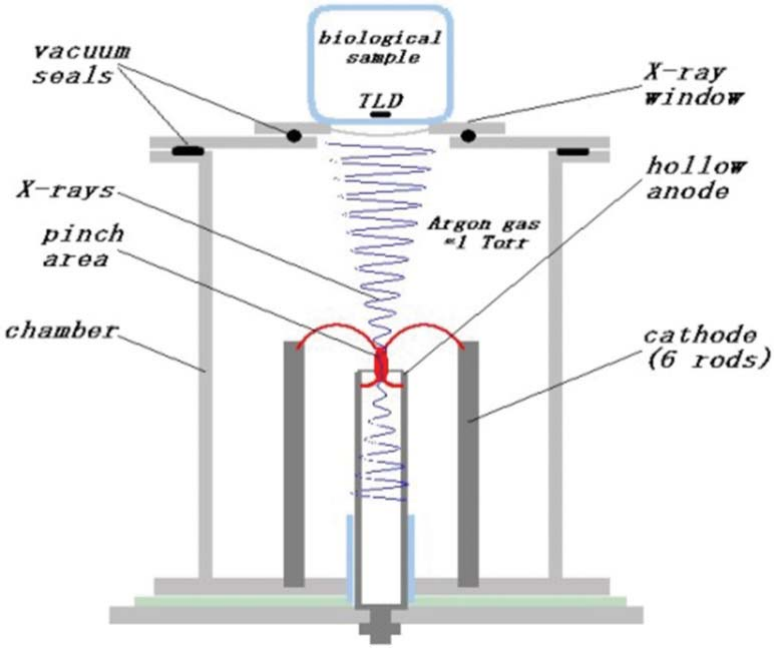
Diagram of the irradiation setup.

A Mather type DPF with an X-ray port on the top was used (Figure 1). As mentioned, this is a pulse-operating high-energy plasma device, which can deliver various types of accelerated particles and photons in a very broad spectral range. In this particular case we use the hard X-rays emitted through the port to investigate their influence on the spores of strain M7. The absorbed doses of X-rays varied in the range of approximately 5–11,000 mSv measured with thermoluminescent dosimeters (TLD).

## Results and discussion

To study the effect of X-ray radiation on endoglucanase activity and total protein (and, respectively, on the overproducer strain), agar-grown cultures and saline spore suspensions were irradiated. In the first part of our experiments, after 120 h of cultivation, the sprouted spores on agar medium were immediately irradiated in DPF.

**Figure 2.**
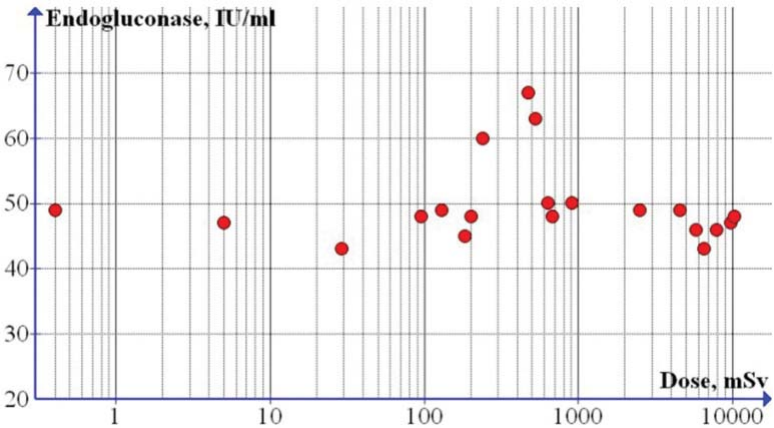
Effect of DPF radiation on endoglucanase activity in batch fermentation: irradiation of 10-day agar-grown cultures.

After 120 h of batch (submerged) cultivation, some enhancement of the endoglucanase activity was observed following irradiation with doses in the range of approximately 250–550 mSv (Figure 2, Table 1). The measured enzyme activity was from 22% up to 36% higher than that observed in non-irradiated spores. The amount of total protein was also increased in the same conditions and at about 471 mSv absorbed dose it was up to 30% higher than that in the non-irradiated spores. Interestingly, the dry biomass measured after 24 h of batch cultivation showed a decrease in the same dose range.

**Table 1.** Effect of DPF radiation on 10-day agar-grown cultures: effect on enzyme activity, total protein in batch fermentation and biomass growth.

Dose absorbed (mSv)	Endoglucanase activity (IU/mL)	Total protein (mg/mL)	Specific endoglucanase activity (U/mL)	Biomass (mg/mL)
Control sample (untreated, 0.4)	49	0.480	102.08	2.96
5	47	0.412	114.08	2.80
29	43	0.409	105.13	3.08
95	48	0.470	102.13	2.96
130	49	0.483	101.45	2.93
184	45	0.439	102.51	2.87
200	48	0.477	100.63	3.01
239	60	0.580	103.45	1.85
471	67	0.627	106.86	2.01
528	63	0.578	109.00	2.27
630	50	0.513	97.47	2.90
675	48	0.491	97.76	3.07
913	50	0.484	103.31	3.10
2493	49	0.489	100.20	3.06
4536	49	0.468	104.70	3.12
5780	46	0.470	97.87	2.89
6566	43	0.429	100.23	2.92
7860	46	0.463	99.35	3.04
9700	47	0.468	100.43	2.96
10,273	48	0.483	99.39	3.01

The second part of our experiment was also conducted with a 10-day culture of *T. reesei*-M7 grown on PDA medium but from the culture so grown we made a spore suspension in saline solution. After that we irradiated this material and made the next cultivation on Mandels medium at the same conditions as the previous experiment.

**Figure 3.**
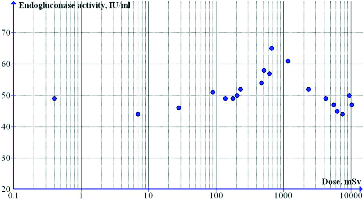
Effect of DPF radiation on endoglucanase activity: irradiation of spore suspension in saline solution.

When the spore suspension was prepared in saline solution, there was also an increase in the endoglucanase activity (Figure 3) and of the total protein (Table 2) as compared to those in the non-irradiated control: a 10%–32% higher endoglucanase activity and 9%–16% higher amount of total protein; as well as a pronounced decrease in dry biomass (Table 2).

**Table 2.** Effect of DPF radiation on a spore suspension in saline solution: effect on enzyme activity, total protein in batch fermentation and biomass growth.

Dose absorbed (mSv)	Endoglucanase activity (IU/mL)	Total protein (mg/mL)	Specific endoglucanase activity (U/mL)	Biomass (mg/mL)
Control sample (untreated, 0,4)	49	0.480	102.08	2.96
7	44	0.402	109.45	2.81
28	46	0.405	113.58	3.11
89	51	0.454	112.33	2.88
137	49	0.467	104.93	2.87
178	49	0.419	116.95	2.92
206	50	0.465	107.53	3.23
232	52	0.476	109.24	2.85
475	54	0.527	102.47	2.56
519	58	0.548	105.84	2.67
618	57	0.533	106.94	2.48
669	65	0.559	116.28	1.88
1174	61	0.552	110.51	2.10
2370	52	0.489	106.34	2.91
4216	49	0.464	105.60	3.18
5601	47	0.479	98.12	2.98
6265	45	0.420	107.14	2.87
7491	44	0.456	96.49	3.15
9542	50	0.488	102.46	2.97
10,346	47	0.468	100.43	3.12

*Trichoderma reesei*-M7 demonstrated high resistance under exposure to high X-ray doses (dose of radiation), with preservation of the viability of the spores (experiments were conducted with up to 20 Sv of absorbed radiation). With the analysis of the endoglucanase activity, the measurement of the dry biomass and the amount of total protein it became evident that for relatively lower doses absorbed (200–1200 mSv), the effect on the micromycetes producer was clearly expressed. With increasing the absorbed dose, on the other hand, measurable changes in the parameters above were not observed. It remains to be further determined whether the observed effect might be considered as a result of direct mutagenic action of high dose rate X-ray radiation on the producer strain.

The obtained results are interesting in view of the fact that severe conditions like extreme radiation doses, very low temperatures, absence of water, etc., are very common in space. It is known that some seeds, spores and even animals (like tardigrades) can survive such conditions. However, in the case with *T. reesei* the exact biochemical and physiological mechanisms underlying such behaviour are not yet clear. Considering the importance of overproducer strains in different fields of biotechnology, the problem is interesting and deserves further investigation.

## Conclusions

This study demonstrated that *T. reesei* strain M7 (overproducer of celluase enzymes) shows high resistance under exposure to high X-ray doses, with preservation of spore viability and endoglucanase activity. The obtained results are interesting, since the exact biochemical and physiological mechanisms underlying resistance to extreme environments are poorly studied in *T. reesei*. Having in mind that overproducer strains that can survive in extreme environments are particularly valuable in different fields of biotechnology, the problem deserves further investigation.
